# ﻿*Polymixiamelanostoma*, a new beardfish from the western Pacific (Teleostei, Polymixiiformes, Polymixiidae)

**DOI:** 10.3897/zookeys.1220.125127

**Published:** 2024-12-09

**Authors:** You-Ci Fan, Yo Su, Chien-Hsiang Lin, Chih-Wei Chang, Hsiu-Chin Lin

**Affiliations:** 1 Department of Oceanography, National Sun Yat-sen University, Kaohsiung, Taiwan; 2 Department of Marine Biotechnology and Resources, National Sun Yat-sen University, Kaohsiung, Taiwan; 3 Department and Graduate Institute of Aquaculture, National Kaohsiung University of Science Technology, Kaohsiung, Taiwan; 4 Biodiversity Research Center, Academia Sinica, Taipei, Taiwan; 5 Marine Ecology and Conservation Research Center, National Academy of Marine Research, Kaohsiung, Taiwan; 6 Graduate Institute of Marine Biology, National Dong Hwa University, and National Museum of Marine Biology and Aquarium, Pingtung, Taiwan; 7 International Doctoral Program of Marine Science and Technology, National Sun Yat-sen University, Kaohsiung, Taiwan

**Keywords:** *COI*, K2P distance, new species, Taiwan, taxonomy

## Abstract

A new species of beardfish, genus *Polymixia*, is described based on three specimens collected in Taiwanese waters and off the Chesterfield Islands of New Caledonia. It can be distinguished from its congeners by the following characters: dorsal-fin rays IV–V, 35–37; gill rakers on outer face of first gill arch 3+1+6=10; scales row between dorsal-fin origin and lateral line vertically 6–8 (S1) and posteriorly 12–14 (S2); pyloric caeca 40; snout rounded, with its surface rough and gelatinous, its tip evidently protrude anterior margin of premaxilla; ctenii on body scales arranged in a wedge-shape, forming three rows; 4^th^ anal-fin spine long, 1.0%–1.2% eye diameter; longer dorsal-fin, 49.6–53.0% SL; blackish oral-branchial cavity. Our maximum-likelihood tree based on mitochondrial *COI* sequences revealed that the new species is distinct from six congeneric species. Our findings indicate the presence of at least six *Polymixia* species in Taiwan, highlighting Taiwan and adjacent areas as a region with the highest diversity of this genus.

## ﻿Introduction

The beardfish genus *Polymixia* Lowe, 1838 comprises the sole surviving clade within the family Polymixiidae (Nelson et al. 2016). Species of *Polymixia* are distinguished by a pair of hyoid barbels attached to the lower jaw, two supramaxillae, villiform teeth on both jaws, vomer, palatine, endopterygoid, and tongue, and the absence of luminous organs (Paxton 1999). They are deep-sea demersal fishes, inhabiting depths of 18–800 m; they are commonly found along the edges of continental shelves, continental slopes, oceanic islands, and near submarine mountains in the Indian, Pacific, and Atlantic oceans (about 45°N to 45°S) (Kotlyar 1986, 1996; Paxton 1999; Hayashi 2013; Nelson et al. 2016; Priede 2017; Anderson 2022).

Currently, 12 species of *Polymixia* are recognized as valid (Caixeta et al. 2024). In the Atlantic Ocean, four species have been documented: *Polymixianobilis* Lowe, 1836, *P.lowei* Günther, 1859, *P.hollisterae* Grande & Wilson, 2021, and *P.carmenae* Caixeta, Oliveira & Melo, 2024. There is one species, *P.fusca* Kotthaus, 1970, in the northwestern Indian Ocean, and two species, *P.berndti* Gilbert, 1905 and *P.busakhini* Kotlyar, 1993, in the Indo-Pacific. Five species occur in the Pacific Ocean, including three species, *P.japonica* Günther, 1877, *P.longispina* Deng, Xiong & Zhang, 1983, and *P.sazonovi* Kotlyar, 1992, in the western Pacific Ocean and two species, *P.yuri* Kotlyar, 1982 and *P.salagomeziensis* Kotlyar, 1991, in the southeastern Pacific Ocean. The Pacific Ocean is the most specious region.

Species of *Polymixia* are similar in general appearances, and some of the diagnostic characters previously used (e.g. Kotlyar 1993, 1996) show high inconsistency. Therefore, genetic characteristics can be useful. However, works on this group including the genetics are limited. Borden et al. (2019), using two mitochondrial and five nuclear loci, revealed nine species-level clades, including five nominal species and four potential new species. Subsequently, only *Polymixia* sp. from Bermuda (*sensu*Borden et al. 2019) has been described as *P.hollisterae* by Grande and Wilson (2021).

Caixeta et al. (2024) used three mitochondrial loci (*COI*, 12S, and 16S) to reconstruct the phylogenetic relationship within *Polymixia* and described a new species, which was previously misidentified as *P.lowei*, from the southwestern Atlantic Ocean. Nonetheless, both the morphological examinations conducted by Borden et al. (2019) and Caixeta et al. (2024) were restricted to a small number of specimens, particularly from the Pacific Ocean (*P.japonica*, *P.berndti*, *P.longispina*, and *P.sazonovi*). Koeda (2019) reported and identified a specimen from southern Taiwan as P.cf.salagomeziensis, which suggests the possibility of additional undescribed *Polymixia* species and highlights the lack of information on Pacific species.

In this study, three specimens of an undescribed *Polymixia* species were collected from southwestern and eastern Taiwan, and the Chesterfield Islands of New Caledonia. This species can be distinguished from its congeners by having more dorsal-fin soft rays, a blackish oral coloration, a long fourth anal-fin spine, and a longer dorsal-fin. Additionally, DNA barcoding provides compelling evidence in support of these specimens as a new species. A detailed species description and comparison with congeners are provided.

## ﻿Materials and methods

All the specimens examined are deposited in the following institutions: Hokkaido University, Laboratory of Marine Zoology, Faculty of Fisheries, The Hokkaido University Museum, Hakodate, Hokkaido, Japan (**HUMZ**), Muséum national d’Histoire naturelle, Paris, France (**MNHN**), and Pisces collection of the National Museum of Marine Biology and Aquarium, Taiwan (**NMMB-P**).

Specimens were fixed in 4% formaldehyde and then transferred to 70% ethanol or 50% isopropanol for permanent preservation. Sagittal otoliths were isolated using “lateral extraction” (Wakefield et al. 2016) and deposited at the Marine Paleontology Lab, Biodiversity Research Center, Academia Sinica, Taipei, Taiwan with catalog numbers CHLOL25609 and CHLOL25610.

### ﻿Morphological analyses

The methodology and terminology employed in this study are based on Kotlyar (1996) with some modifications, including forehead height was determined from the upper margin of the orbit to the center of the interorbital space, and the length of the longest dorsal-fin spine was measured additionally. Moreover, the numbers of pseudobranchial filaments were recorded.

All counts of paired elements were conducted on the left side unless damaged, in which case the right side was used and noted. The number of gill rakers represents developed rakers on the outer side of the first arch (upper limb + angle + lower limb). Scale rows were defined and counted as follows: scale rows between dorsal-fin origin and lateral line counted vertically (S1) and counted posteriorly (S2); scale rows between lateral line and anal-fin origin (S3). Vertebral counts were obtained using x-radiographs taken by a digital radiograph machine located at the National Museum of Marine Biology and Aquarium, Pingtung, Taiwan.

Measurements were taken using 150 mm digital calipers rounding to the nearest 0.1 mm, except for lengths longer than 150 mm, which were taken using a regular ruler rounding to the nearest 1 mm. Data of measurements are expressed as ratios or percentages to standard length (SL) or head length (HL), except where noted. The description of otoliths follows Lin and Chang (2012).

### ﻿Phylogenetic analyses

Before fixation in the formaldehyde, tissue samples were taken from the base of the anal fin or dorsal fin of each specimen, fixed in 95% ethanol, and stored at room temperature. DNA extraction method followed the protocol of the Tissue & Cell Genomic DNA Purification Kit (Biokit, Taiwan). A fragment of mitochondrial cytochrome c oxidase I gene (*COI*) was amplified and sequenced using the published primer pair FishF1 (5′-TCA ACC AAC CAC AAA GAC ATT GGC AC-3′) and FishR1 (5′-TAG ACT TCT GGG TGG CCA AAG AAT CA-3′) from Ward et al. (2005).

Sequences generated in this study were submitted to GenBank (Benson et al. 2012) with accession numbers PP556533 to PP556549 (Table 1). Other sequences of *Polymixia* were retrieved from GenBank and BOLD systems (Ratnasingham and Hebert 2007) (Table 1). Additionally, sequences of *Gadusmorhua* (MT893167.1 and MT455539.1) were selected as the outgroup. Sequences were aligned by the ClustalW model (Thompson et al. 1994) using the default settings in Geneious v. 8.1.9 (Kearse et al 2012).

**Table 1.** *COI* sequences of *Polymixia* used for genetic analyses in this study. Sequences marked with an asterisk (*) were generated for this study and others were retrieved from GenBank (Benson et al. 2012) or BOLD system (Ratnasingham and Hebert 2007).

Species	Accession numbers
*Polymixiamelanostoma* **sp. nov.**	PP556538*, PP556540*
*P.japonica*	PP556536*, PP556546*, PP556547*, PP556548*, ZOSKT1538-16, GBMTG154-16, ON398672.1
*P.fusca*	KU375762.1
*P.lowei*	SCAFB121-07, SCAFB144-07, SCFAC471-06, GBGCA12516-15, BCOLL237-06, BCOLL238-06, BCOLL239-06, BCOLL240-06, BCOLL241-06, ANGBF40917-19, ANGBF40918-19, UKFBI1117-08, GBMTG226-16, MLIII513-08
*P.carmenae*	OR660087.1, OR660087.1
*P.longispina*	PP556537*, PP556539*, PP556541*, PP556543*, PP556545*, PP556549*, FOAM828-11, FOAM829-11, FOAO209-14, FOAO212-14, FOAO213-14, FOAO214-14, FOAO2368-20, ZOSKT314-16, GBMIN94135-17
*P.berndti*	PP556533*, PP556534*, PP556535*, PP556542*, PP556544*, FOAF463-07, FOAF464-07, FOAF772-07, FOAN778-11, TZMSC367-05, TZMSC405-05, TZMC409-05, DSFSF399-09, DSFSF720-09
*Gadusmorhua*	MN893167.1, MT455539.1

A hypothesized phylogenetic tree was reconstructed using the maximum-likelihood method, employing the Hasegawa-Kishino-Yano (HKY) model (Hasegawa et al. 1985), and 1,000 bootstrap pseudoreplicates were performed by the software MEGAX (Kumar et al. 2018). Genetic distances were calculated also using the K2P model (Kimura 1980).

## ﻿Results

### ﻿Genus Polymixia

Taxon classificationAnimaliaPolymixiiformesPolymixiidae

Lowe, 1836

BEDA8859-8AB9-5B1E-A0E1-16CC287D44EF

#### Type species.

*Polymixianobilis* Lowe, 1836.

### ﻿ Polymixia melanostoma sp. nov.

Taxon classificationAnimaliaPolymixiiformesPolymixiidae

7B2FEDD0-F810-5E46-99D8-487F4F59B26E

https://zoobank.org/783ECB16-7601-4FE9-AAC6-907EE440BB65

**Figure 1.**
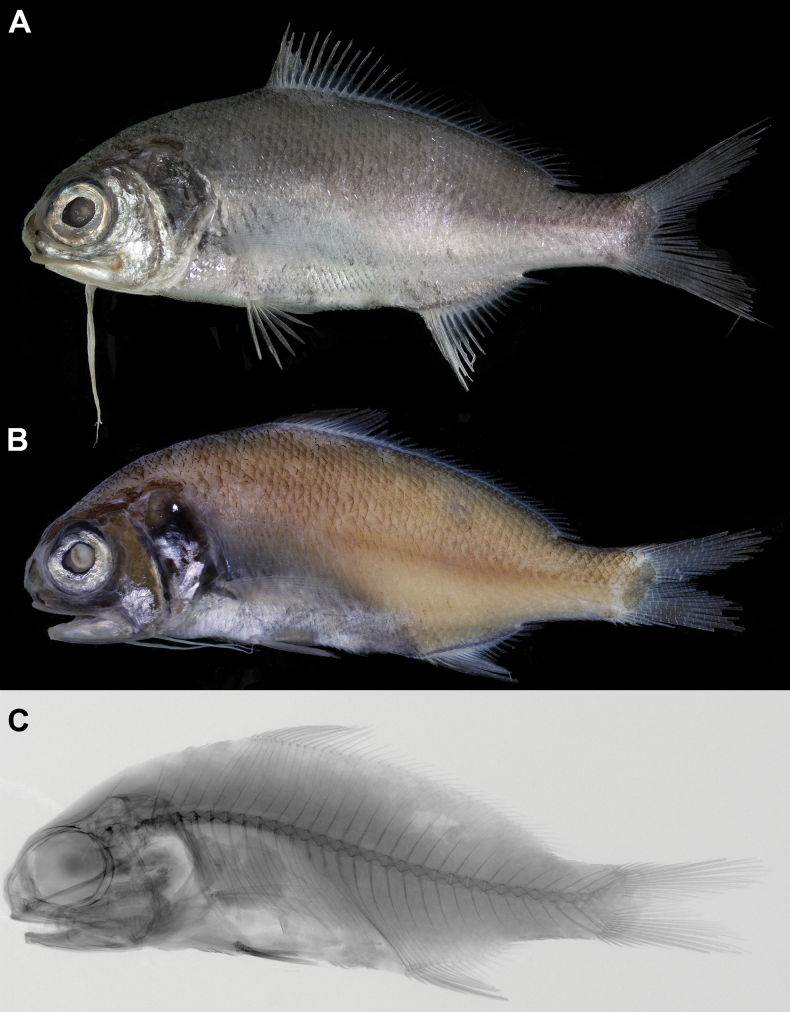
*Polymixiamelanostoma* sp. nov., holotype, NMMB-P39587, 110.2 mm SL**A** fresh **B** preserved **C** x-radiograph.

**Figure 2.**
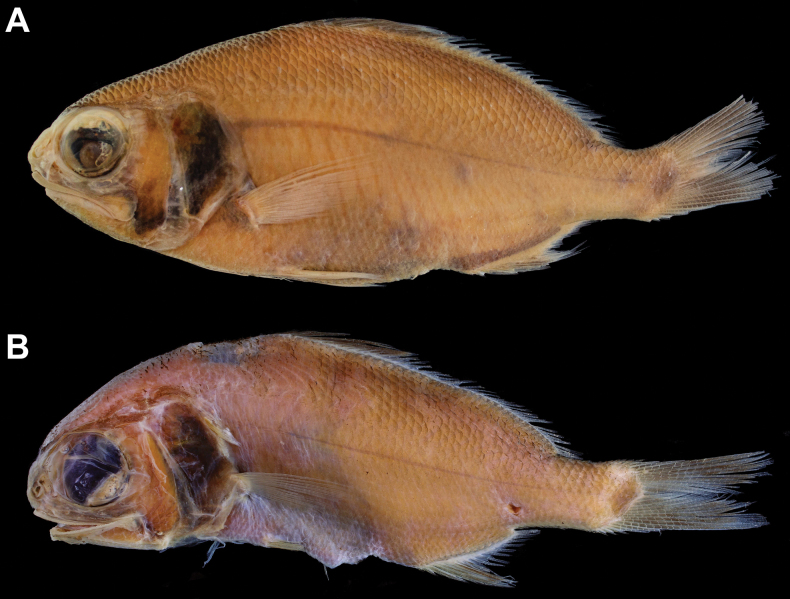
Preserved paratypes of *Polymixiamelanostoma* sp. nov. **A**MNHN 2014-2291, 153 mm SL**B** NMMB-P39588, 122.5 mm SL.

**Figure 3.**
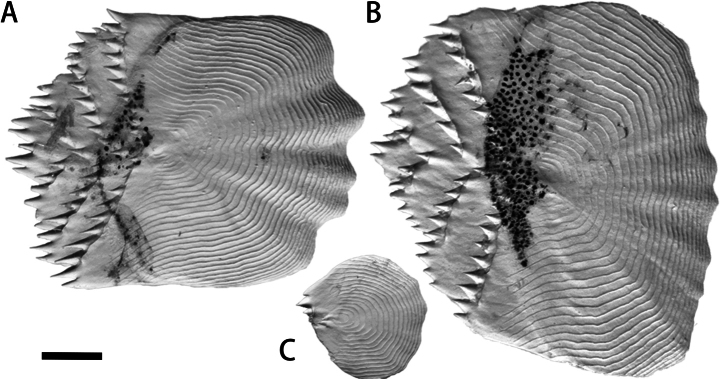
Right-side scales of *Polymixiamelanostoma* sp. nov., holotype, NMMB-P39587, 110.2 mm SL**A** scale on caudal peduncle **B** scale on anterior dorsal-fin base **C** scale on isthmus. Scale bar: 1 mm.

**Figure 4.**
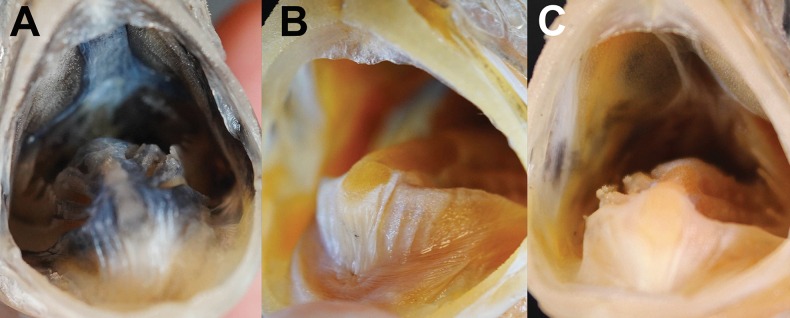
Close-up image of oral-cavity coloration of preserved **A***Polymixiamelanostoma* sp. nov., holotype, NMMB-P39587, 110.2 mm SL**B***P.longispina*, NMMB-P39585, 128.5 mm SL**C***P.japonica*, NMMB-P39573, 104.9 mm SL. Figure not to scale.

**Figure 5.**
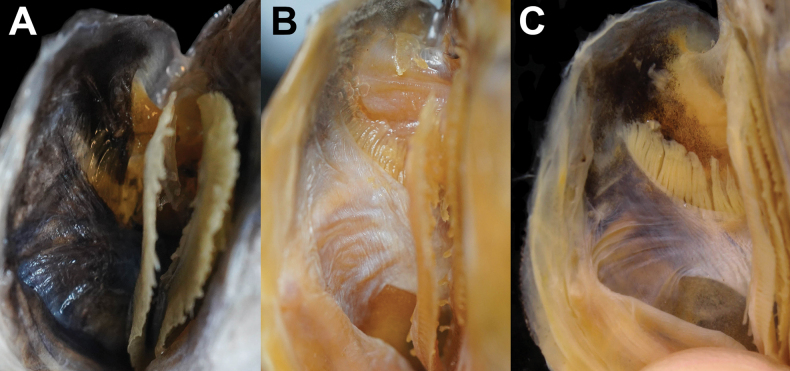
Close-up image of branchial-cavity coloration of preserved **A***Polymixiamelanostoma* sp. nov., holotype, NMMB-P39587, 110.2 mm SL**B***P.longispina*, NMMB-P39585, 128.5 mm SL**C***P.japonica*, NMMB-P39573, 104.9 mm SL. Figure not to scale.

**Figure 6.**
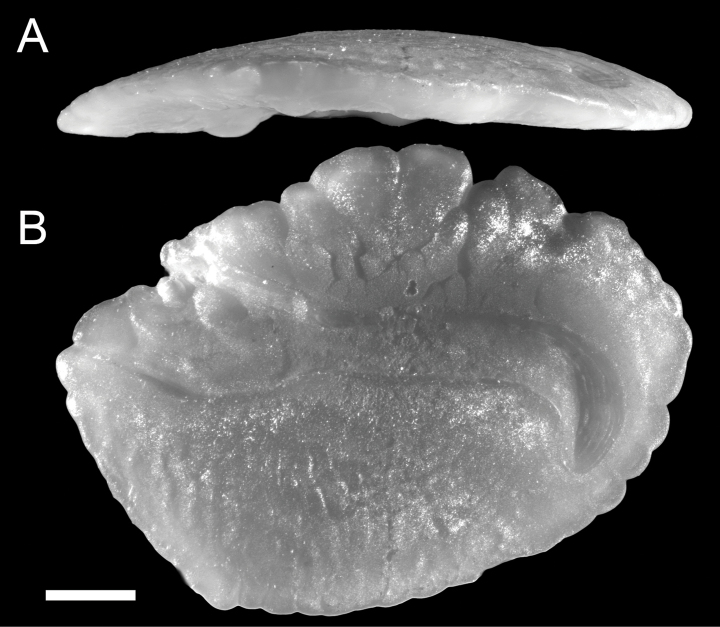
Right sagittal otolith of *Polymixiamelanostoma* sp. nov., paratype (CHLOL25610, from NMMB-P3988, 122.5 mm SL) **A** ventral face **B** inner face. Scale bar: 1 mm.

**Figure 7.**
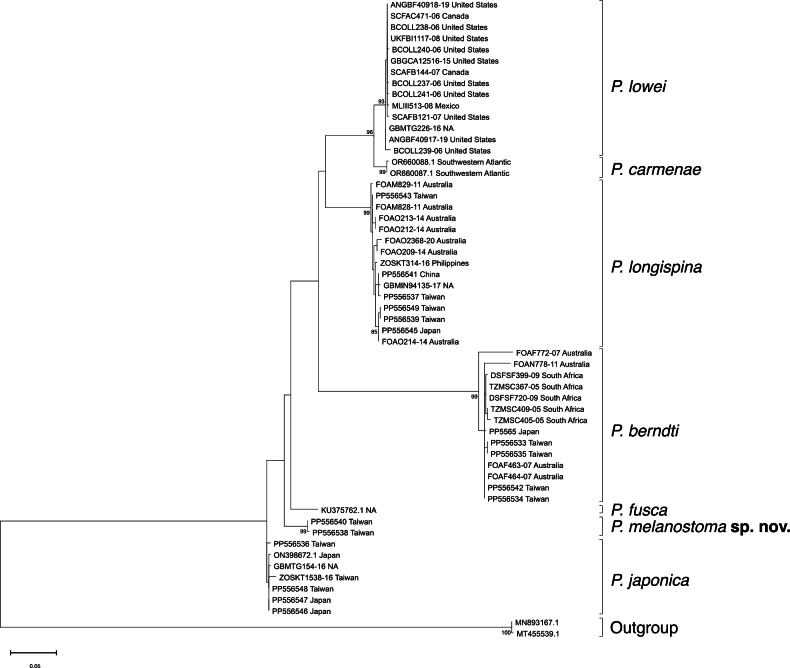
Maximum-likelihood tree reconstructed using *COI* sequence. The number next to each node represents bootstrap value >75%. Scale bar at bottom corner represents the number of substitutions per unit length on the tree.

Figs 1 , 2 , 3 , 4A , 5B , 6 , 7 ; Tables 1 , 2 , 3 , 4 New English name. Black-mouth beardfish New Chinese name. 黑口鬚銀眼鯛

Polymixia japonica (non Günther): Li 2010: 12–13 (in part, specimens were mixed with P.japonica).

#### Type specimens.

***Holotype***: Taiwan • NMMB-P39587 (110.2 mm SL); northern South China Sea, Pingtung, off Dong-gang fishing port; ca 22°22'22"N, 120°27'34"E; 27 January 2023; Y. Su leg.; in bottom trawl; *COI*: PP556538.

***Paratypes*** (*n*=2; SL 122.5–153.0 mm): Taiwan • 1 specimen; NMMB-P39588 (SL 122.5 mm); Taitung, off Chenggong fishing port; ca 23°05'52.93"N, 121°22'43.05"E; 11 September 2009; purchased by C.-W. Chang; hook and line; *COI*: PP556540. – New Caledonia • 1 specimen; MNHN 2014-2291 (153 mm SL); Coral Sea, Chesterfield Islands; 21°10'2.40"S, 158°37'24.01"E; 765–778 m deep; 11 October 2005; EBISCO, at st. CP2545.

#### Etymology.

The specific name *melanostoma* is a combination of Greek *melano* and *stoma*, meaning “black mouth”, in reference to its unique black oral cavity.

#### Diagnosis.

*Polymixiamelanostoma* sp. nov. differs from its congeners in having the following combination of characters: dorsal-fin rays IV–V, 35–37; gill rakers on the outer face of first gill arch 3+1+6=10; S1 6–8; S2 12–14; pyloric caeca 40; snout rounded, with surface rough and gelatinous, its tip evidently protrude anterior margin of premaxilla; ctenii on body scales arranged in wedge shape, forming three rows; 4^th^ anal-fin spine long, 1.0–1.2 in eye diameter; dorsal-fin long, 49.6–53.0% SL; oral-branchial cavity, not including the underside of tongue, black.

#### Description.

Meristic and morphometric data are provided in Tables 1, 2. Data below are for the holotype, followed by a range of paratypes in parentheses, except where indicated.

**Table 2.** Meristic and morphological characters of *Polymixiamelanostoma* sp. nov. and three similar sympatric congeners. Abbreviation: NT, non-types.

	*P.melanostoma* sp. nov.		*P.japonica*	*P.berndti*	*P.longispina*
	Holotype	Paratype (*n*=2)	NT (*n*=20)	NT (*n*=39)	NT (*n*=29)
Dorsal-fin rays	V, 35	IV–V, 36–37	IV–VI, 31–34	IV–VI, 28–31	V, 28–32
Pectoral-fin rays	17	15–16	15–17	13–17	15–17
Pelvic-fin rays	i+6	i+6	i+6	i+6	i+6
Anal-fin rays	IV, 14	IV, 14–16	IV–V, 14–16	III–IV, 13–17	IV, 13–16
Gill rakers	3+1+6=10	3+1+6=10	3–4+1+7–9=11–14	3–4+1+7–8=11–13	4+1+6–9=11–14
Pseudobranchial filaments	27	33	26–35	21–32	20–25
Pyloric caeca	40	—	37–56 (*n* = 2)	41–55 (*n* = 7)	26–30 (*n* = 3)
Vertebrae	12+17=29	12+17=29	12+16–17=28–29	12–13+17=29–30	12+17=29
Lateral line scales	35	35–39	30–34	27–36	29–36
S1	6	7–8	6–8	5–7	4–7
S2	12	14	11–16	8–11	8–11
S3	15	16	13–17	11–16	10–16
Snout tip	Protruded		Not protruded	Protruded	Not or slightly protruded
Oral cavity	Black		White or partly black	White	White
Branchial cavity	Black		Black	White	White
Scales ctenii distribution	Wedge		Wedge	Vertical	Vertical

Dorsal-fin rays V, 35 (IV–V, 36–37); pectoral-fin rays 17 (15–16); pelvic-fin rays i+6 (i+6); anal-fin rays IV, 14 (IV, 14–16); principal caudal-fin rays 9+9=18 (9+9=18), uppermost and lowermost rays unbranched; procurrent caudal-fin rays 5 (6) dorsally and 5 (5) ventrally; lateral-line scales 35 (35–39); S1 6 (7–8); S2 12 (14); S3 15 (16, n=1); gill rakers on the outer face of first gill arch 3+1+6=10 (3+1+6=10); pseudobranchial filaments 27 (33); pyloric caeca 40; branchiostegal rays 7 and only posterior 4 visible. Vertebrae 12+17=29 (12+17=29); supraneural and pterygiophore insertion formula: 0/0//0/1+1/1+1/1+1/1+1/1+1/1+1/1+1/1+1/1+1/1+1/1+1+1/1+1/1+1/1+1/1+1/1+1/1+1+1/1+1+1/2 (0/0//0/1+1/1+1/1+1/1+1/1/1+1/1+1/1+1+1/1+1+/1+1+1/1+1/1+1/1+1+1/1+1/1+1+1/1+1/1+1+1/1+2, n=1).

Body rather slender, longer than deep, depth at dorsal-fin origin 2.8 (2.5–3.0) in SL. Head large, its length 2.9 (2.8–2.9) in SL; from snout to forehead rising gently; upper profile in front of dorsal fin slightly concave; forehead narrow, its width 6.1 (7.5–7.6) in HL; eyes large, 2.8 (2.6–3.0) in HL; snout rounded, its surface rough and gelatinous with several small bumps, tip protruding anteriorly from premaxilla, its length 6.1 (5.8–6.1) in HL; space between eyes convex and rather narrow, interorbital width 3.0 (3.1–3.4) in HL.

Mouth large, posterior end of maxilla extending distinctly beyond vertical through posterior margin of eye. Nostrils close together but separated by narrow membranes, both immediately in front of anterior margin of eye and below horizontal through of eye; posterior nostril much larger than anterior one. No knob at symphysis of dentaries. Two supramaxillae, with anterior one triangular and posterior one rectangular, rounded posteriorly, not covering posterior portion of maxilla; postventral corner of maxilla exposed. Posterior-ventral margin of preopercle serrated with tip slightly pointed. Lower-jaw barbels, length 1.2 (1.0–1.1) in HL, its end exceeds pelvic-fin origin.

Most portions of lateral and medial surfaces of premaxilla and dentary covered with villiform teeth. Palatine and ectopterygoid with narrow band of villiform teeth; vomer with an oblong patch of villiform teeth. Endopterygoid with a large patch of villiform teeth. Gill rakers rod-shaped, somewhat laterally compressed, with villiform teeth on inner surfaces; those in outer row of the first arch longest; rakers on inner row of the first arch and both inner and outer rows of the second to third arches short, forming bumps; those on the fourth arch forming bumps; small tooth patches forming bumps, present on midline of all four outer arches. Villiform, teardrop-like tooth patches present on the fifth ceratobranchial. Small villiform teeth patch on the second pharygobranchial forming oval patches. Large, teardrop-like tooth patches on the third pharyngobranchial.

Body covered with firmly attached ctenoid scales; ctenii on body scales arranged in a wedge shape, forming three rows (Fig. 3A). Isthmus with ctenoid scales; gular region naked, without scales.

Dorsal fin long, 2.0 (1.9–2.0) in SL. Dorsal-fin spines progressively longer posteriorly, length of the longest spine 0.9 (0.8–0.9) in eye diameter; outer margin of soft rays slightly concave; the first soft ray longest, and gradually shorter posteriorly. Anal-fin spines progressively longer posteriorly, length of the longest spine rather long, 1.2 (1.0–1.2) in eye diameter; outer margin of soft rays slightly concave; first soft ray longest. Pectoral fin short, 1.6 (1.5–1.8) in HL, its tip not reaching lateral line when adpressed to body. Pelvic fin without spine, its end slightly exceeds through pectoral-fin end vertical. Caudal-fin concave and pointed. Pyloric caeca pale, unbranched.

#### Size.

Moderately small species. The biggest specimen examined was 153.0 mm SL.

#### Coloration.

When fresh, body silvery, with dorsum slightly dusky (Fig. 1A). Tip of anteriormost nine dorsal-fin soft rays black, forming distinct spot (paratype). Anal-fin rays pale, second to fourth spines and anteriormost five soft rays with few black pigmentations. Caudal fin grayish. Pelvic fin pale, with black pigmentations on first, fifth, and seventh rays.

Body color, when preserved, similar to when fresh, slightly yellowish (Figs 1B, 2). Snout semitransparent. Oral cavity, including dorsal surface of tongue black (Fig. 4A). Underside of tongue pale. Inner side of opercle and peritoneum black (Fig. 5A). Tip of lower jaw without black spots.

#### Otolith.

The sagittal otolith was taken from NMMB-P39588 (122.5 mm SL) and measured 6.8 mm in length (Fig. 6). Otolith rhomboidal (length/height ratio 1.36), moderately thick (Fig. 6A). Dorsal margin gently raised, lobed, highest at central; ventral margin slightly crenulated, regularly curved, deepest slightly anterior to central; posterior margin blunt, crenulated, not extending posteriorly; anterior margin triangular with incised notch; rostrum short and broad; antirostrum pointed; inner face convex; outer face slightly concave; sulcus centrally positioned, well divided into ostium and cauda; ostium wider than cauda, short, oblong, nearly filled with colliculum; cauda elongated, strongly curved at posterior with tip directing ventrally; cristae well developed; dorsal depression shallow, just above crista superior.

#### Distribution.

This species is known from specimens collected from southwestern and eastern Taiwan and the Chesterfield Islands of New Caledonia, suggesting a broad distribution in the western Pacific Ocean. Inhabits at depth down to 778 m (based on MNHN 2014-2291).

#### Genetic analysis.

The phylogenetic tree of *Polymixia* was reconstructed by the maximum-likelihood method with HKY+G+I (Hasegawa et al. 1985) nucleotide substitution models. The monophyly of *P.melanostoma* sp. nov. specimens is strongly supported by a bootstrap value of 99% (Fig. 7).

The pairwise genetic distance analysis with the K2P model reveals an average interspecific distance of 4.4–14.5% between *P.melanostoma* sp. nov. and the other six congeneric species (Table 4). The shortest genetic distance is with *P.japonica*, while the farthest is with *P.berndti*.

#### Comparison.

*Polymixiamelanostoma* sp. nov. can be distinguished from 10 of the 12 congeneric species in having fewer gill rakers (3+1+6=10 vs 3–13+1+6–13=11–24 in *P.lowei*, *P.japonica*, *P.berndti*, *P.fusca*, *P.yuri*, *P.longispina*, *P.salagomeziensis*, *P.busakhini*, *P.sazonovi*, and *P.carmenae*; Table 2; Kotlyar 1996; Caixeta et al. 2024). Among these congers, only *P.nobilis* has a count that overlaps with *P.melanostoma* sp. nov. Borden et al. (2019) suggested that *P.nobilis* might not only occur in the Atlantic Ocean but also the Pacific. However, in comparison to the description and data provided by Kotlyar (1996), *P.melanostoma* sp. nov. differs from *P.nobilis* in having fewer pyloric caeca (40 vs 108 in *P.nobilis*), fewer S2 (12–14 vs 15–16), different vertebral formula (12+17=29 vs 14+15=29), a longer head (34.2–35.4% SL vs 30.8–32.3% SL), a longer dorsal fin (49.6–53.0% SL vs 41.3–44.3% SL), and a more protruding snout (vs slightly protruding).

Although the number of gill rakers for *Polymixiahollisterae* was not documented by Grande and Wilson (2021), *P.melanostoma* sp. nov. differs from *P.hollisterae* in having more dorsal-fin rays (IV, 35–37 vs V, 31–32), more pyloric caeca (40 vs 30), and higher numbers of S1 (6–8 vs 5), S2 (12–14 vs 10), and S3 (15–16 vs 12–14).

Compared with the species co-occurring in Taiwan (*Polymixiajaponica*, *P.longispina*, and *P.berndti*), *P.melanostoma* sp. nov. exhibits meristic counts overlapping with *P.japonica* and shares distinctive characteristics, including a black branchial cavity (Fig. 5) and ctenii on scales distributed in a wedge shape (Fig. 3). It differs from *P.japonica* in having fewer number of gill rakers (3+1+6=10 vs 3–4+1+7–9=11–14; Table 2), more dorsal-fin rays (IV–IV, 35–37 vs IV–VI, 31–34; Table 2), a longer dorsal fin (49.6–53.0% SL vs 42.5–48.3% SL; Table 3), a fully black oral cavity (vs partly black; Fig. 4, Table 2), and a more protruding snout (vs not protruding; Table 2).

**Table 3.** Morphometric data for *Polymixiamelanostoma* sp. nov. and three similar sympatric congeners. Abbreviations: A, anal-fin; D, dorsal-fin; NT, non-types; P, pectoral-fin; V, pelvic-fin.

	*P.melanostoma* sp. nov.			*P.japonica*		*P.berndti*		*P.longispina*	
	Holotype	All types (*n* = 3)		NT (*n* = 20)		NT (*n* = 39)		NT (*n* = 30)	
SL (mm	110.2	128.5 (110.2–152)		98.2 (88.7–107.1)		106.3 (62.0–173)		104.8 (62.4–135.5)	
% SL		Mean(range)	S.D.	Mean(range)	S.D.	Mean(range)	S.D.	Mean(range)	S.D.
Head length	34.3	34.6 (34.2–35.4)	0.7	32.6 (30.1–34.9)	1.4	34.8 (32.8–37.7)	1.8	34.3 (30.2–36.5)	1.5
Body depth	35.6	36.2 (33.3–39.6)	3.2	35.4 (30.3–41.4)	2.9	35.9 (32.7–40.6)	1.8	38.9 (33.9–42.2)	1.9
Eye diameter	12.1	12.4 (12.0–13.2)	0.7	11.2 (8.8–12.5)	0.9	11.2 (9.7–12.9)	0.8	11.1 (8.9–12.9)	0.8
Interorbital	11.6	11.0 (10.5–11.6)	0.5	10.3 (8.8–12.3)	0.9	10.6 (8.7–12.4)	1.0	11.1 (9.4–12.7)	0.7
Upper-jaw length	17.8	18.6 (17.8–19.1)	0.7	17.4 (16.1–19.2)	0.9	18.9 (17.2–20.3)	0.6	17.9 (15.3–19.4)	0.9
Lower-jaw length	15.3	16.3 (15.3–17.0)	0.9	15.8 (14.4–19.5)	1.3	17.4 (16.0–19.8)	0.9	16.6 (12.9–21.7)	1.3
Snout length	5.6	5.8 (5.6–6.1)	0.3	6.0 (5.1–7.0)	0.5	5.9 (4.3–7.0)	0.7	7.1 (3.7–8.8)	1.3
Postorbital length	17.0	17.5 (17.0–18.3)	0.7	17.1 (15.3–18.8)	1.1	19.1 (16.9–20.8)	1.0	18.4 (17.3–19.5)	0.6
Forehead height	5.6	4.9 (4.6–5.6)	0.6	5.6 (4.2–7.6)	0.9	5.8 (3.9–10.7)	1.2	7.4 (3.8–10.7)	1.4
Predorsal length	46.3	46.6 (46.3–47.1)	0.4	48.4 (45.4–51.6)	1.8	51.2 (48.5–54.7)	1.7	55.1 (49.1–58.5)	2.7
Prepectoral length	33.8	34.1 (33.8–34.5)	0.4	32.5 (29.5–34.6)	1.1	34.5 (32.3–37.4)	1.2	35.1 (31.3–37.3)	1.6
Prepelvic length	38.4	40.5 (38.4–43.0)	2.3	39.2 (36.4–42.0)	1.3	41.4 (35.8–43.7)	1.7	41.4 (35.3–44.4)	2.0
Preanal length	66.0	68.5 (66.0–71.5)	2.8	68.3 (65.1–71.3)	1.6	72.9 (67.3–77.7)	2.0	70.0 (63.3–75.7)	2.6
D length	49.6	51.1 (49.6–53.0)	1.7	45.3 (42.5–48.3)	1.5	42.8 (40.7–47.1)	1.2	43.2 (40.7–46.4)	1.5
D height	13.7	14.7 (13.7–16.6)	1.7	14.7 (12.1–17.0)	1.2	17.1 (13.1–20.1)	1.6	15.1 (12.7–19.3)	1.8
Longest D spine	10.5	10.7 (10.1–11.5)	0.8	13.1 (11.3–15.0)	1.1	10.6 (5.7–15.0)	2.0	12.8 (9.5–19.7)	2.4
A length	18.9	17.6 (16.8–18.9)	1.1	19.3 (17.9–21.2)	0.9	17.5 (15.0–21.6)	1.5	18.5 (16.5–20.7)	1.1
A height	13.5	14.1 (13.5–15.0)	0.8	12.8 (11.5–15.8)	1.2	12.2 (7.2–15.2)	1.5	13.9 (12.2–19.0)	1.6
Longest A spine	14.9	14.0 (12.0–15.2)	1.7	12.9 (10.3–15.2)	1.2	10.1 (7.7–13.5)	1.5	15.1 (12.6–22.9)	2.6
D–P length	27.6	28.0 (23.6–33.0)	4.7	29.2 (25.8–32.7)	2.0	29.0 (17.8–31.5)	2.2	31.1 (26.9–34.6)	1.6
D–V length	35.5	36.2 (33.3–39.8)	3.3	36.4 (32.9–41.4)	2.7	36.3 (33.3–39.3)	1.6	39.0 (35.9–42.6)	1.7
D–A length	44.7	45.8 (44.3–48.3)	2.2	43.9 (40.9–47.6)	2.0	42.5 (37.9–47.4)	1.7	45.9 (42.4–50.6)	1.7
P–A length	28.2	28.4 (26.1–30.8)	2.3	31.2 (28.7–33.8)	1.2	33.4 (29.4–38.8)	2.1	30.3 (26.6–33.7)	1.8
P–V length	12.1	12.8 (12.1–13.6)	0.7	11.8 (10.1–13.8)	1.1	11.4 (7.1–15.5)	1.7	12.9 (11.6–14.9)	0.6
P length	22.1	21.9 (19.5–24.1)	2.3	18.3 (14.8–21.4)	1.6	20.5 (18.2–22.2)	1.0	20.4 (17.8–23.2)	1.5
V length	14.1	14.1 (12.9–15.1)	1.1	12.5 (10.7–14.6)	1.1	13.9 (12.3–15.7)	0.9	13.0 (11.3–14.6)	0.8
Caudal-peduncle length	11.0	12.4 (11.0–13.4)	1.2	13.5 (11.7–16.4)	1.3	12.7 (10.2–16.2)	1.8	12.9 (10.4–15.5)	1.1
Caudal-peduncle height	11.0	10.7 (10.6–11.0)	0.2	10.9 (10.0–11.9)	0.6	10.1 (9.0–12.3)	0.7	10.7 (9.7–13.4)	0.8
Caudal-fin length	27.4	28.6 (27.4–29.7)	1.7	28.8 (24.9–33.8)	2.4	27.6 (24.6–30.3)	1.7	28.4 (24.6–38.1)	2.4
Barbel length	29.4	31.8 (29.4–34.5)	2.6	29.3 (26.1–35.6)	2.6	28.8 (23.6–32.8)	2.2	33.9 (22.6–40.0)	5.5

**Table 4.** Interspecific genetic distance of *COI* sequences of *Polymixia* species calculated with Kimura-2-parameter model (Kimura 1980). The number in the first row correspond to the species names in the first column. Values are presented as percentage (%).

		1	2	3	4	5	6	7
1	*P.melanostoma* **sp. nov.**	–	–	–	–	–	–	–
2	*P.japonica*	4.4	–	–	–	–	–	–
3	*P.fusca*	5.3	5.1	–	–	–	–	–
4	*P.longispina*	8.8	8.3	9.8	–	–	–	–
5	*P.carmenae*	9.7	9.4	9.1	8.9	–	–	–
6	*P.lowei*	10.0	9.7	8.9	8.8	2.7	–	–
7	*P.berndti*	14.5	16.0	13.2	15.3	14.5	14.1	–

Additionally, *P.melanostoma* sp. nov. is also similar to *P.longispina* in having a long fourth anal-fin spine (12.0–15.2% SL, mean 14.0 vs 12.6–22.9% SL, mean 15.1; Table 3) but differs from it in having less gill rakers (3+1+6=10 vs 4+1+6–9=11–14; Table 2), more dorsal-fin rays (IV, 35–37 vs V, 28–32; Table 2), more pyloric caeca (40 vs 26–30; Table 2), more S2 (12–14 vs 8–11; Table 2), a shorter predorsal length (46.3–47.1% SL vs 49.1–58.5% SL; Table 3), a longer dorsal-fin (49.6–53.0% SL vs 40.7–46.4% SL; Table 3), a black oral cavity (vs white; Fig. 4; Table 2), a black branchial cavity (vs white; Fig. 5; Table 2), a more protruding snout (vs slightly protruding; Table 2), and the wedge-shaped distribution of ctenii on scales (vs vertical; Fig. 3; Table 2).

*Polymixiamelanostoma* sp. nov. differs from *P.berndti* in having less gill rakers (3+1+6=10 vs 3–4+1+7–8=11–13; Table 2), more dorsal-fin rays (IV, 35–37 vs IV–VI, 28–31; Table 2), more S2 (12–14 vs 8–11; Table 2), a shorter predorsal length (46.3–47.1% SL vs 48.5–54.7% SL; Table 3), a longer dorsal-fin (49.6–53.0% SL vs 40.7–47.1% SL; Table 3), a black oral cavity (vs white; Fig. 4; Table 2), a black branchial cavity (vs white; Fig. 5, Table 2), and the wedge-shaped distribution of ctenii on scales (vs vertical; Fig. 3, Table 2).

## ﻿Discussion

Previous studies of *Polymixia* mostly focused on morphological characters (Kotlyar 1986, 1996), while knowledge of genetic distinctions within *Polymixia* is limited. Although Borden et al. (2019) and Caixeta et al. (2024) utilized several genetic loci to reconstruct their phylogenetic relationships, they included only a few sequences of each species. Our study retrieved a total of 55 *COI* sequences, including 17 newly obtained sequences, for the reconstruction of a maximum-likelihood tree. This tree revealed seven distinct species-level clades (Fig. 7). Among these clades, the new species, *P.melanostoma* sp. nov. shows a close relation to *P.japonica* with the lowest genetic distance of 4.4% (Table 4). *P.melanostoma* sp. nov. and *P.japonica* share several morphological characteristics: higher numbers of dorsal-fin rays (≥31), higher numbers of S2 (≥11), a black branchial cavity, and ctenii on scales distributed in wedge shape.

Based on the sampling locations retrieved from the BOLD system and GenBank, the distribution of the other six *Polymixia* species are updated as follows (Fig. 7): *P.japonica* is only found in the northwestern Pacific Ocean (Taiwan and Japan), presenting a comparatively smaller geographic distribution when compared to its congeners. *Polymixialongispina* is distributed in the northwestern Pacific Ocean (Taiwan, China, and Japan) and in the eastern Indian Ocean (off Western Australia). *Polymixialowei* is found in the Western Atlantic Ocean off the eastern United States, Mexico, and Canada. *Polymixiacarmenae* is distributed in southwestern Atlantic Ocean. *P.berndti* is widely distributed in the Indian Ocean (South Africa and off Western Australia) and in the northwestern Pacific Ocean (Taiwan and Japan). However, the absence of sequences from the type locality, Hawaii, of *P.berndti* raises questions about whether this clade represents true *P.berndti*. Lastly, the sampling location for the sequence of *P.fusca* in the study remains unknown, casting uncertainty on its representation and distribution.

Taiwan exhibits a remarkable diversity of *Polymixia* species. Koeda (2019) documented four species and one undescribed species in Taiwan, namely *P.japonica*, *P.berndti*, *P.longispina*, *P.sazonovi*, and P.cf.salagomeziensis. The current study recognizes four species, *P.japonica*, *P.berndti*, *P.longispina*, *P.sazonovi*, and describes an additional new species, *P.melanostoma* sp. nov. To summarize, these findings suggest that at least six *Polymixia* species have been distributed in Taiwan, highlighting Taiwan as a region with the highest diversity of *Polymixia*.

### ﻿Comparative materials

*Polymixiajaponica* (20 specimens, 88.7–107.1 mm SL): HUMZ 207076, 104.2 mm SL, Isshiki Fish Market Aichi, Japan, 14 February, 2010; HUMZ 207259, 91.4 mm SL, Numazu fish market, Shizuoka, Japan, 16 February, 2010; HUMZ 226776, 2 specimens, 88.7–89.0 mm SL, 36°29.03"N, 140°57.73"E, off Hitachi, Ibaraki, Japan, 3 November, 2015; HUMZ 231889, 89.3 mm SL, Nishiura fish market, Aichi, Japan, 15 March 2021, *COI*: PP556546; HUMZ 231090, 91.9 mm SL, 36°28'55.20"N, 140°58'44.40"E, off Hitachi, Ibaraki, Japan, 12 November 2019, *COI*: PP556547; NMMB-P39573, 10 specimens, 96.7–105.5 mm SL, Dong-gang, Pingtung, southwestern Taiwan, 6 January 2023, *COI*: PP556536, PP556548; NMMB-P3954, 99.7 mm SL, Dong-gang, Pingtung, southwestern Taiwan, 6 January 2023; NMMB-P39575, 94.6 mm SL, Dong-gang, Pingtung, southwestern Taiwan, 19 February 2023; NMMB-P31551, 107.1 mm SL, Dong-gang, Pingtung, southwestern Taiwan, 31 March 2018; NMMB-P16414, 96.6 mm SL, Dong-gang, Pingtung, northeastern Taiwan, 21 February 2012.

*Polymixiaberndti* (39 specimens, 62.0–173 mm SL): HUMZ 226763, 67.7 mm SL, 36°29'2.40"N, 140°57'43.20"E, off Hitachi, Ibaraki, Japan, 3 November 2015, *COI*: PP556544; NMMB-P39578, 8 specimens, 86.2–134.2 mm SL, Dong-gang, Pingtung, southwestern Taiwan, 6 January 2023, *COI*: PP556533–PP556535; NMMB-P39579, 88.1 mm SL, Dong-gang, Pingtung, southwestern Taiwan, 6 January 2023; NMMB-P39580, 3 specimens, 74.7–130.8 mm SL, Dong-gang, Pingtung, southwestern Taiwan, 14 January 2023; NMMB-P39582, 3 specimens, 85.1–90.5 mm SL, Dong-gang, Pingtung, southwestern Taiwan, 3 November 2022; NMMB-P39581, 15 specimens, 61.9–107.2 mm SL, Dong-gang, Pingtung, southwestern Taiwan, 19 February 2023; NMMB-P29357, 2 specimens, 154–155 mm SL, Nan-Fang-ao, Yilan, northeastern Taiwan, 5 April 2018; NMMB-P36406, 2 specimens, 148.3–151 mm SL, Dong-gang, Pingtung, southwestern Taiwan, 4 March 2022; NMMB-P16154, 155 mm SL, Hualien, eastern Taiwan, 27 May 2010; NMMB-P8730, 173 mm SL, Nanwan, Pingtung, southern Taiwan, 23 June 2005; NMMB-P 35954, 164 mm SL, Kinmen, western Taiwan, 17 July 2020; NMMB-P39583, 148.6 mm SL, Nan-fang-ao, Yilan, northeastern Taiwan, 5 July 2010; NMMB-P39967, 67.8 mm SL, Dong-gang, Pingtung, southwestern Taiwan, 21 August 2023, *COI*: PP556542.

*Polymixialongispina* (30 specimens, 62.4–135.5 mm SL): HUMZ 229136, 36°55'44.40"N, 141°32'34.80"E, off Iwaki, Fukushima, Japan, 3 November 2017, *COI*: PP556545; NMMB-P39584, 74.2 mm SL, Dong-gang, Pingtung, southwestern Taiwan, 6 January 2023, *COI*: PP556537; NMMB-P9908, 110.7 mm SL, Cheng-gung, Taitung, eastern Taiwan, 4 June 2009; NMMB-P9902, 135.5 mm SL, Cheng-gung, Taitung, eastern Taiwan, 4 June 2009; NMMB-P9904, 112.0 mm SL, Cheng-gung, Taitung, eastern Taiwan, 4 June 2009; NMMB-P36407, 88.9 mm SL, Dong-gang, Pingtung, southwestern Taiwan, 12 March 2022; NMMB-P9060, 2 specimens, 64.6–68.6 mm SL, Dong-gang, Pingtung, southwestern Taiwan, 13 June 2008; NMMB-P9909, 117.4 mm SL, Cheng-gung, Taitung, eastern Taiwan, 4 June 2009; NMMB-P9903, 131.1 mm SL, Cheng-gung, Taitung, eastern Taiwan, 11 September 2009; NMMB-P9910, 122.3 mm SL, Cheng-gung, Taitung, eastern Taiwan, 4 June 2009; NMMB-P35563, 3 specimens, 62.4–69.2 mm SL, Dongsha, Kaohsiung, Southern Taiwan, 18 April 2021; NMMB-P36409, 2 specimens, 63.5–66.0 mm SL, Dong-gang, Pingtung, southwestern Taiwan, 9 April 2022; NMMB-P39585, 15 specimens, 99.1–129.4 mm SL, Cheng-gung, Taitung, eastern Taiwan, 4 June 2009, *COI*: PP556539; NMMB-P39586, 2 specimens, 109.4–126.1 mm SL, Cheng-gung, Taitung, eastern Taiwan, 11 September 2009; NMMB-P39969, 66.4 mm SL, Hainan, China, 18 July 2022, *COI*: PP556541; NMMB-P39970, 64.9 mm SL, Dong-gang, Pingtung, southwestern Taiwan, 3 April 2023, *COI*: PP556543.

## Supplementary Material

XML Treatment for Polymixia

XML Treatment for Polymixia melanostoma

## ﻿Additional information

### Author ORCIDs

You-Ci Fan https://orcid.org/0009-0008-3937-9026

Yo Su https://orcid.org/0000-0002-3576-9229

Chien-Hsiang Lin https://orcid.org/0000-0002-9843-9729

Chih-Wei Chang https://orcid.org/0000-0002-0659-9582

Hsiu-Chin Lin https://orcid.org/0000-0001-8116-0484

### Data availability

All of the data that support the findings of this study are available in the main text.
